# Speed and accuracy instructions affect two aspects of skill learning differently

**DOI:** 10.1038/s41539-022-00144-9

**Published:** 2022-10-22

**Authors:** Teodóra Vékony, Claire Pleche, Orsolya Pesthy, Karolina Janacsek, Dezso Nemeth

**Affiliations:** 1 Lyon Neuroscience Research Center (CRNL), INSERM U1028, CNRS UMR5292, Université Claude Bernard Lyon 1, Lyon, France; 2 Département d’Études Cognitives, École Normale Supérieure, Université PSL, 75005 Paris, France; 3 Doctoral School of Psychology, ELTE Eötvös Loránd University, Budapest, Hungary; 4 Institute of Psychology, ELTE Eötvös Loránd University, Budapest, Hungary; 5 Centre of Thinking and Learning, Institute for Lifecourse Development, School of Human Sciences, Faculty of Education, Health and Human Sciences, University of Greenwich, London, UK; 6 Brain, Memory and Language Research Group, Institute of Cognitive Neuroscience and Psychology, Research Centre for Natural Sciences, Budapest, Hungary

**Keywords:** Human behaviour, Learning and memory

## Abstract

Procedural learning is key to optimal skill learning and is essential for functioning in everyday life. The findings of previous studies are contradictory regarding whether procedural learning can be modified by prioritizing speed or accuracy during learning. The conflicting results may be due to the fact that procedural learning is a multifaceted cognitive function. The purpose of our study is to determine whether and how speed and accuracy instructions affect two aspects of procedural learning: the learning of probability-based and serial-order-based regularities. Two groups of healthy individuals were instructed to practice on a cued probabilistic sequence learning task: one group focused on being fast and the other on being accurate during the learning phase. The speed instruction resulted in enhanced expression of probability-based but not serial-order-based knowledge. After a retention period, we instructed the participants to focus on speed and accuracy equally, and we tested their acquired knowledge. The acquired knowledge was comparable between groups in both types of learning. These findings suggest that different aspects of procedural learning can be affected differently by instructions. However, only momentary performance might be boosted by speed instruction; the acquired knowledge remains intact. In addition, as the accuracy instruction resulted in accuracy near ceiling level, the results illustrate that response errors are not needed for humans to learn in the procedural domain and draw attention to the fact that different instructions can separate competence from performance.

## Introduction

The ability to acquire motor, perceptual, and social skills enables us to function effectively in everyday life. Many skills are initially learned with external instructions; for example, the driving instructor usually gives the student specific instructions on how to handle the car, or the trainer tells the athlete to focus on accuracy or speed for a particular movement. Although many different instructions can be given during skill learning, for instance, to break down the to-be-learned elements into chunks^1^ or to focus explicitly on particular regularities^2,3^, speed and accuracy instructions are frequently used during real-life skill learning as well as in laboratory experiments. Additionally, it is also essential to understand the effect of such instructions because neuroscience studies are often interpreted in conjunction with behavioral results, thus, the potential effects of instructions also play an important role in how we interpret neuroscientific results on skill learning. Here, we aim to investigate how emphasizing speed or accuracy influences different aspects of skill learning.

Scientific interest has increased to reveal the optimal circumstances to excel in skill acquisition, an essential element of which is procedural learning^4–6^. Studies investigating skill acquisition in sports have found that novice learners benefit from accurate instructions, whereas proceduralized skills of experts become more enhanced under speed constraints^7–9^. Similarly, Hoyndorf and Haider^10^ have found accuracy strategy to impair the expression of implicit skill acquisition compared to speed instruction; however, learning did occur under accuracy instructions. The study of Barnhoorn and colleagues^11^ has found that speed instruction enhances the representations of repeating explicit sequences, while more accurate responses lead to a faster selection of responses via better stimulus-response associations. Studies above indicate that speed and accuracy instructions can affect skill learning.

Contrarily, in our previous work, we have shown that skill learning is not necessarily boosted by speed constraints: we have found that procedural knowledge can be equally acquired and maintained under speed and accuracy instructions^12^. Importantly, procedural learning is a multifaceted cognitive function^13–15^: possibly, the different results were due to a lack of sufficient separation between learning mechanisms, with different proportions of them contributing to the outcome. This raises the question of which aspect of procedural learning is more influenced by speed or accuracy instruction. In this follow-up study, we sought to fill this gap by exploring the effects of speed and accuracy instructions on two essential aspects of procedural learning: acquiring and retrieving probability-based and serial-order-based regularities.

Procedural learning encompasses learning of different forms of regularities that can be distinguished on both conceptual and methodological levels^13–16^. One major aspect of procedural learning is the ability to learn the order of a series of repeated events (with some embedded noise, in probabilistic sequences, or without noise, in deterministic sequences). We refer to this type of learning as serial-order-based learning. Another aspect of procedural learning is the ability to learn frequency or probability-based short-range associations between elements of the sequence, hereafter referred to as probability-based learning. From a theoretical perspective, it is important to note that both forms of learning can be considered statistical learning: probability-based learning refers to the acquisition of second-order transitional probabilities that are less than one, whereas serial-order-based learning refers to the acquisition of transitional probabilities that are equal to one.

While both types of learning are based on input statistics, they measure independent features of the input structure. Previous studies have shown that these two aspects of learning are even characterized by distinct neural mechanisms^14,16–18^ and developmental trajectories^19^. Moreover, it has been shown that while probability-based learning typically occurs incidentally (implicitly) and relatively rapidly, resulting in robust representations, serial-order-based learning may occur either incidentally or intentionally and take a longer time to develop^14,16^. There is no need for a previously built-up representation for probability-based learning, as only the detection of local statistical structures is required, leading to learning that occurs incidentally. A serial-order-based learning approach, on the other hand, involves a more global and complex representation of sequence structure determined by interactions of multiple events across space and time and is, therefore, more akin to intentional, goal-directed learning. We hypothesize that the more incidental, rapid learning of probability-based regularities could be more easily manipulated by instructions, and based on previous findings indicating better procedural learning under speed instructions^7,8,10^, we expect speed instructions to boost the expression of the probability-based aspect of procedural learning.

Our previous results using the non-cued version of the Alternating Serial Reaction Time (ASRT) task showed that neither the expression of procedural knowledge nor the extent of the acquired knowledge was affected by speed or accuracy instructions^12^. Here, to further elaborate on these results, we aimed to identify which aspect of procedural learning played a role in this previous null result. Using the non-cued version of the task, the probability- and serial-order-based aspects of learning would only become distinguishable at the behavioral level after 7–8 45 min-long sessions^20^, as serial-order-based learning develops much slower than probability-based learning^15^. To overcome this issue, we used the cued version of the ASRT task^19^. The cueing allows us to dissociate these two aspects of procedural learning after a few blocks of practice, bringing them into the same time frame of acquisition. In this four-choice visuomotor task, predetermined (pattern) trials alternate with randomly chosen trials. This alternating structure results in some chunks of stimuli being more probable than others. Serial-order-based learning can be measured by the difference between elements appearing as part of the predetermined pattern vs. appearing randomly but with equal appearance probabilities. Probability-based learning can be measured by contrasting the random trials with different probabilities (see more details in the Methods section).

To test how speed and accuracy instructions affect the expression of probability-based and serial-order-based knowledge, we instructed participants to practice the cued version of the ASRT task focusing on speed or accuracy (Different Instruction Phase). After a short break, participants’ acquired probability- and serial-order-based knowledge were tested again, this time, with a focus on both speed and accuracy. Our main goal was to determine whether (1) speed/accuracy instructions affect probability-based vs. serial-order-based knowledge differently, (2) the instructions affect the expression of knowledge (Different Instruction Phase) and the acquired knowledge (Same Instruction Phase) equally. Although our study is exploratory, we hypothesized that probability-based learning processes would be more affected by instructions than serial-order-based learning.

## Results

### Did the two groups perform equally before learning (practice phase)?

To ensure that the potential differences in learning were not due to pre-existing differences in baseline RT and accuracy between the groups, we compared the median RT and accuracy between the two groups during the practice session. No differences were found in RT (*U* = 294, *p* = 0.88, *r*_RB_ = 0.03, *BF*_01_ = 3.03) or accuracy (*U* = 297.5, *p* = 0.82, *r*_RB_ = 0.04, *BF*_01_ = 3.43).

### Did the instructions change overall reaction times and accuracy?

To test whether overall reaction time (RT) in the Different Instruction Phase differed between groups (i.e., whether the speed/accuracy instruction had the expected effect on response tendencies), a mixed-design ANOVA with the within-subject factor of Epoch (Epoch 1 vs. Epoch 2 vs. Epoch 3 vs. Epoch 4) and the between-subject factor of Group (Speed Group vs. Accuracy Group) was run with median RT as the dependent variable. A gradual decrease in RTs was observed over the course of the task, irrespective of trial type [main effect of Epoch, *F*(1.65, 75.71) = 47.23, *p* < 0.001, η_*p*_^2^ = 0.51, *BF*_exclusion_ < 0.01]. Response times were significantly faster in the Speed Group than in the Accuracy Group [M_Accuracy Group_ = 464 ms ± 13.22 SE, M_Speed Group_ = 348 ms ± 13.22 SE, main effect of Group: *F*(1,47) = 38.13, *p* < 0.001, η_*p*_^2^ = 0.45, *BF*_exclusion_ < 0.01]. The Epoch × Group interaction was not significant, *F*(1.65, 75.71) = 0.39, *p* = 0.64, η_*p*_^2^ = 0.01, *BF*_exclusion_ = 2.89.

A similar mixed-design ANOVA was performed with accuracy as the dependent variable to test whether accuracies were different between the groups in the Different Instruction Phase. Accuracy decreased over the course of learning, irrespective of trial type [main effect of Epoch: *F*(1.23, 56.57) = 9.12, *p* = 0.002, η_*p*_^2^ = 0.17, *BF*_exclusion_ < 0.001]. The Accuracy Group were more accurate than the Speed Group [M_Accuracy Group_ = 98.6% ± 2.2 SE, M_Speed Group_ = 83.3% ± 2.2 SE, main effect of Group: *F*(1,53) = 24.18, *p* < 0.001, η_*p*_^2^ = 0.35, *BF*_exclusion_ < 0.001]. The change in accuracy over the course of learning was different between the two groups as a gradual decrease in accuracy was only detectable in the Speed Group [Epoch × Group interaction: *F*(1.23, 56.57) = 6.16, *p* = 0.01, η_*p*_^2^ = 0.12, *BF*_exclusion_ = 0.01].

The RTs and accuracies were also compared during the Same Instruction Phase. The Speed Group remained slightly faster (M_Accuracy Group_ = 355 ms ± 7.13 SE, M_Speed Group_ = 331 ms ± 7.68 SE, *U* = 396.5, *p* = 0.02, *r*_RB_ = 0.39, *BF*_01_ = 0.34) and less accurate (M_Accuracy Group_ = 96.3% ± 0.4 SE, M_Speed Group_ = 92.3 % ± 1.6 SE, *U* = 430.5, *p* = 0.003, *r*_RB_ = 0.50, *BF*_01_ = 0.09) than the Accuracy Group after the change of the instructions.

Taken together, speed and accuracy instruction affected the response tendencies of the participants: the Speed Group was faster and less accurate, while the Accuracy Group was slower and more accurate in the Different Instruction Phase. As slight differences remained in the Similar Instruction Phase, further analyses of both phases were performed on standardized values [learning score of the given epoch/median RT of the given epoch].

### Did the instructions affect the learning of probability-based regularities in the Different Instruction Phase?

**Fig. 1:**
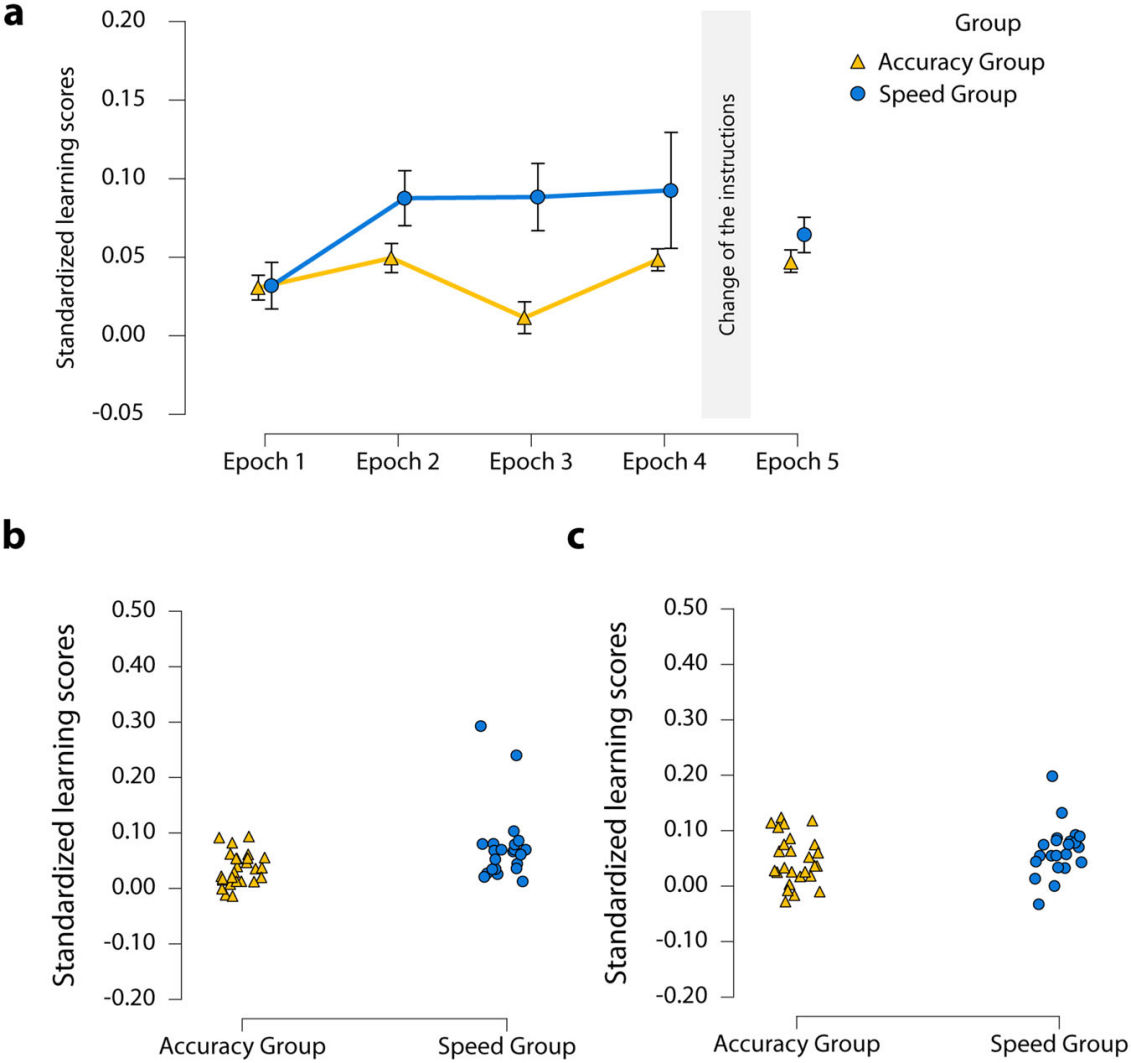
Learning of probability-based regularities. **a** The dynamics of learning of probability-based regularities with accuracy or speed instructions. The *y*-axis represents the standardized learning scores [(random low-probability trials − random high-probability trials in the given epoch)/median RT of the given epoch], and the *x*-axis of the five epochs (the first four are of the Different Instruction Phase, and the fifth one is of the Same Instruction Phase). The Accuracy Group is presented with yellow, while the Speed Group with blue color. Error bars represent the standard error of the mean. In the Different Instruction Phase, the Speed Group shows an advantage of learning, but it disappears in the Same Instruction Phase. **b** Individual data of the significant main effect of Group of probability-based learning in the Different Instruction Phase. Triangles and dots represent the individual data points. **c** Individual data of the lack of significant main effect of Group of probability-based learning in the Same Instruction Phase. Triangles and dots represent the individual data points.

The learning of probability-based regularities was measured by the difference between random high-probability and random low-probability trials (standardized by the median RT of the corresponding epoch). A mixed-design ANOVA was run on the probability-based learning scores of the Different Instruction Phase with the within-subject factor of Epoch (Epoch 1 vs. Epoch 2 vs. Epoch 3 vs. Epoch 4) and the between-subject factor of Group (Accuracy Group vs. Speed Group). Overall, Speed Group showed larger learning scores compared to the Accuracy Group [M_Accuracy Group_ = 0.04 ± 0.01 SE, M_Speed Group_ = 0.08 ± 0.01 SE, main effect of Group: *F*(1, 46) = 7.64, *p* = 0.008, η_*p*_^2^ = 0.14, *BF*_exclusion_ = 0.48]. No main effect of Epoch was found, *F*(1.80, 82.79) = 2.21, *p* = 0.12, η_*p*_^2^ = 0.05, *BF*_exclusion_ = 3.11, and the interaction between Epoch and Group was non-significant, *F*(1.80, 82.79) = 1.57, *p* = 0.22, η_*p*_^2^ = 0.03, *BF*_exclusion_ = 3.46 (Fig. 1).

### Did the instructions affect knowledge of probability-based regularities in the Same Instruction Phase?

In the Same Instruction Phase, standardized probability-based learning scores were compared between the groups. Contrary to the results of the Different Instruction Phase, no difference was found between groups in the Same Instruction Phase, *U* = 221, *p* = 0.18, *r*_RB_ = −0.23, *BF*_01_ = 2.20 (Fig. 1).

### Did the instructions affect the learning of serial-order-based regularities in the Different Instruction Phase?

**Fig. 2:**
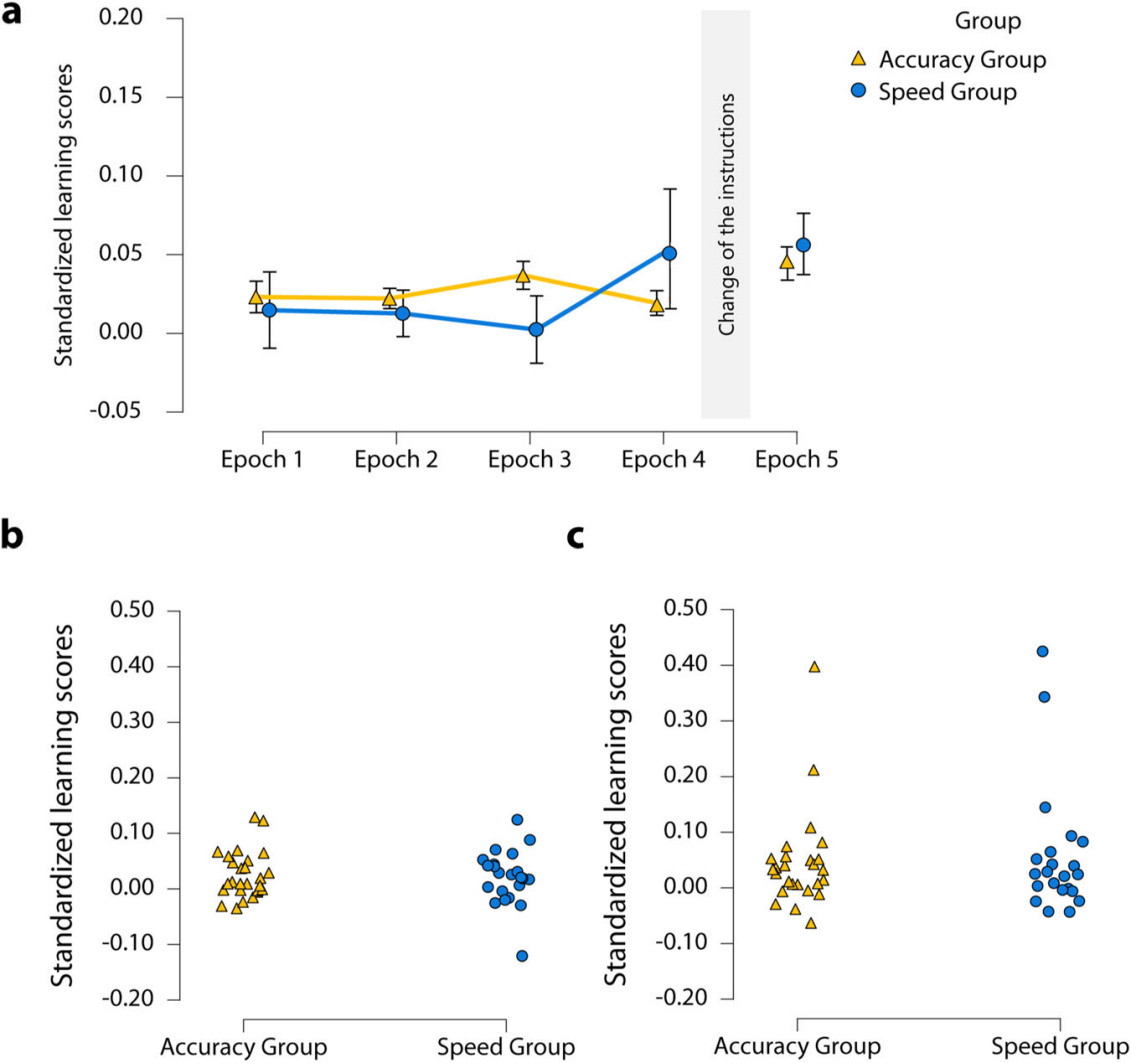
Learning of serial-order-based regularities. **a** The dynamics of learning of serial-order-based regularities with accuracy or speed instructions. The *y*-axis represents the standardized learning scores [(random high-probability trials − pattern high-probability trials in the given epoch)/median RT of the given epoch], and the *x*-axis of the five epochs (the first four are of the Different Instruction Phase, and the fifth one is of the Same Instruction Phase). The Accuracy Group is presented with yellow, while the Speed Group with blue color. Error bars represent standard error of the mean. Both groups show equal learning in both phases. **b** Individual data of the lack of significant main effect of Group of serial-order-based learning in the Different Instruction Phase. Triangles and dots represent the individual data points. **c** Individual data of the lack of significant main effect of Group of serial-order-based learning in the Same Instruction Phase. Triangles and dots represent the individual data points.

The learning of serial-order-based regularities was quantified by the difference in RTs between random high-probability vs. pattern high-probability trials (standardized by the median RT of the corresponding epoch). A mixed-design ANOVA was run on the standardized serial-order-based learning scores of the Different Instruction Phase with the within-subject factor of Epoch (Epoch 1 vs. Epoch 2 vs Epoch 3 vs. Epoch 4) and the between-subject factor of Group (Accuracy Group vs. Speed Group). The two groups showed similar level of serial-order-based learning in the Different Instruction Phase [M_Accuracy Group_ = 0.03 ± 0.01 SE, M_Speed Group_ = 0.02 ± 0.01 SE, main effect of Group: *F*(1, 46) = 0.12, *p* = 0.74, η_*p*_^2^ = 0.003, *BF*_exclusion_ = 7.38]. No main effect of Epoch was found, *F*(1.51, 69.30) = 0.48, *p* = 0.57, η_*p*_^2^ = 0.01, *BF*_exclusion_ = 32.98, and the interaction between Epoch and Group was non-significant, *F*(1.51, 69.30) = 1.23, *p* = 0.29, *η*_*p*_^2^ = 0.03, *BF*_exclusion_ = 138.65, indicating that the trajectory of learning was also similar (Fig. 2).

### Did the instructions affect knowledge of serial-order-based regularities in the Same Instruction Phase?

Standardized serial-order-based learning scores were compared between the groups in the Same Instruction Phase. Similar to the results of the Different Instruction Phase, no significant difference was found between the two groups in the Same Instruction Phase, *U* = 295, *p* = 0.86, *r*_RB_ = 0.03, *BF*_01_ = 3.49 (Fig. 2).

### Did the instructions affect the ability to report explicitly on the sequence?

**Fig. 3:**
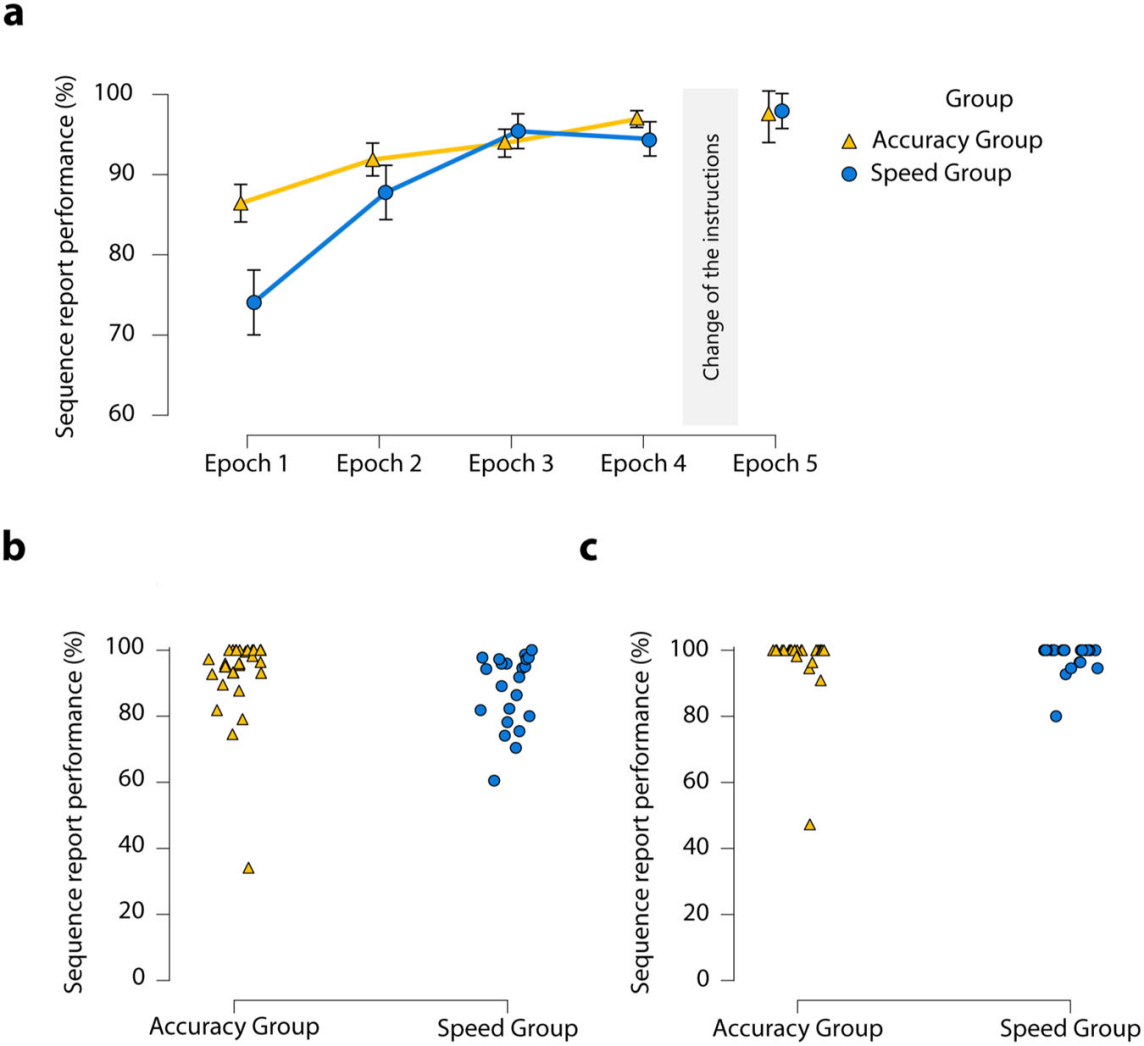
Post-block sequence report performance. **a** Dynamics of post-block sequence report performance. The *y*-axis indicates the mean sequence report performance in percentage. The *x*-axis shows the epochs (Epoch 1–4: Different Instruction Phase, Epoch 5: Same Instruction Phase). The yellow line indicates the Accuracy Group, and the blue line the Speed Group. Both groups were able to report on the sequence. No significant group difference was found in either phase; however, on a trend-level, the Accuracy Group performed better in the first epoch. The error bars represent standard error of the mean. **b** Individual data of the lack of significant main effect of Group of the main post-block sequence report performance in the Different Instruction Phase. Triangles and dots represent the individual data points. **c** Individual data of the lack of significant main effect of Group of the main post-block sequence report performance in the Same Instruction Phase. Triangles and dots represent the individual data points.

We performed a mixed-design ANOVA on the explicit sequence report scores of the *Different Instruction Phase* with the within-subject factor of Epoch (Epoch 1 vs. Epoch 2 vs Epoch 3 vs. Epoch 4) and the between-subject factor of Group (Accuracy Group vs. Speed Group). Sequence report performance improved over time as revealed by the main effect of Epoch, *F*(2.22, 102.09) = 17.19, *p* < 0.001, η_*p*_^2^ = 0.27, *BF*_exclusion_ < 0.001. Overall, the performance of the two groups was similar [main effect of Group: *F*(1, 46) = 1.44, *p* = 0.24, *η*_*p*_^2^ = 0.03, *BF*_exclusion_ = 1.64]. However, the interaction between the Epoch and Group factors approached significance [Epoch × Group interaction: *F*(2.22, 102.09) = 2.94, *p* = 0.05, η_*p*_^2^ = 0.06, *BF*_exclusion_ = 0.81]. This trend-level effect was due to the fact that Accuracy Group performed better on sequence reports in the first epoch [M_Accuracy Group_ = 86.4% ± 2.3 SE, M_Speed Group_ = 74% ± 2.4 SE]; however, they achieved similar performance by the last epoch of the Different Instruction Phase [M_Accuracy Group_ = 96.9% ± 2.6 SE, M_Speed Group_ = 94.5% ± 2.6 SE] (Fig. 3).

In *the Same Instruction Phase*, the two groups performed similarly in terms of explicit sequence report scores, M_Accuracy Group_ = 97.2%, M_Speed Group_ = 97.9%, *U* = 309, *p* = 0.53, *r*_RB_ = 0.08 *BF*_01_ = 2.88 (Fig. 3).

## Discussion

The aim of our study was to reveal the effects of speed and accuracy instructions on two essential aspects of procedural learning. To this end, healthy young adults were tested on a cued version of a probabilistic sequence learning task, which enabled us to quantify the effects of speed and accuracy instructions on the learning of probability-based and serial-order-based regularities. Participants performed the task with the instruction to focus on speed or accuracy during the task (Different Instruction Phase). Following a short break, participants were tested again, but this time, they had to focus on speed and accuracy equally (Same Instruction Phase). We found that the performance during the learning of probability-based information was improved under speed instruction, whereas performance during the learning of serial-order-based regularities was not affected by the two different strategies. It is important to emphasize that only the expression of knowledge was affected by the instructions. When the acquired knowledge was tested (with instructions to focus on speed and accuracy equally), equal probability-based and serial-order-based knowledge were revealed irrespective of whether participants focused on speed or accuracy during the initial learning.

Our previous results using the non-cued version of the probabilistic sequence learning task (in the non-cued version, the pattern and random trials are not differentiated by visually distinct stimuli, and participants are not informed that every second element is part of a repeating pattern) showed that neither the expression of knowledge nor the acquired knowledge was affected by speed or accuracy instructions^12^. In the current study, similar to the results of the non-cued version of the task, acquired knowledge was resistant to instructions, thus, both probability-based and serial-order-based regularities were learned successfully under speed and accuracy instructions. The non-cued version of the task could not easily disentangle the probability- and serial-order-based aspects of learning, because using the non-cued version, these two aspects of learning will only become visible after 7–8 45 min-long sessions of practice^20^. Based on the results of the current study (i.e., that expression of the knowledge of probabilities can be affected by instructions), the lack of performance difference using the non-cued version might have been due to the serial-order-based learning component, which proves to be resistant to speed and accuracy instructions. Our finding that the expression of the knowledge of probability-based regularities is better under speed instructions, but the acquired knowledge is not affected is somewhat congruent with the findings of Hoyndorf and Haider^10^. In their study, better learning performance was found under speed instructions; however, similarly to our results, this was attributed only to performance effects, that is, to the alterations in performance rather than to the direct effect on learning per se.

Alternatively, the difference between our findings with the non-cued and cued versions of the probabilistic learning task could be explained by the fact that memorizing sequence elements requires divided attention, which may affect the outcome of probability-based learning. However, this is unlikely because (1) it has been shown previously that there is no difference between implicit and cued versions in terms of probability-based learning^21^, and (2) learners with both accuracy and speed instructions were able to report the sequence to the same extent. Therefore, we can conclude that speed and accuracy instructions differently affect the knowledge expression of probability- and serial-order-based information.

Given the slope of the learning curve in our study, in contrast to the explanation of Hoyndorf and Haider^10^, it is more likely that the speed instruction improved the expression of the knowledge of probabilities than that the accuracy instruction worsened it; however, this advantage is no longer measurable without the help of the speed instruction. This finding draws attention to an important problem in the measurement of learning. The majority of cognitive studies measure learning in a single context and draw conclusions about brain-behavior relationships based on the expression of knowledge or “momentary performance”, i.e., the temporary fluctuation of behavior^22–25^. However, the measured momentary performance is not always equivalent to the acquired knowledge (competence) that could be measured in the long term. Acquired knowledge (competence) and knowledge expression (performance) can differ due to many factors such as fatigue, manipulation of inter-stimulus intervals, practice, latent learning, or overlearning of the skill^26,27^. Our study showed that competence and performance can differ, especially when evaluating probability-based learning. Therefore, using only a single session to evaluate learning might be problematic when we intend to describe the long-term knowledge of probability-based regularities, which serves as the base of many learning, memory, and decision-making tasks (e.g., Gluck, Shohamym & Myers^28^). If contextual factors influence the measured performance, and we use these scores to make conclusions on brain-behavior relationships, then it can lead to wrong conclusions easily. We recommend taking into account the possible differences between measured competence and performance when designing learning studies.

Our study showed that participants learning with accuracy instructions acquired stable probability-based and serial-order-based knowledge despite minimizing motor (response) errors during initial learning. Although in terms of probability-based knowledge, the speed instruction showed an advantage during the initial learning, the acquired knowledge was comparable with both instructions for both probability- and serial-order-based learning. Based on the theory that the brain functions as a Bayesian inference machine^29^, our results are especially intriguing as they can seemingly contradict findings that errors facilitate learning^30^. In our brain, associations between events are formed by continuously, adjusting the estimated probability distribution, which is referred to as the prior. If a prediction error is made, the prior should be updated according to the new probabilistic structure^29^. Our study - similarly to our findings with the non-cued version of the probabilistic sequence learning task^12^ and other sequence learning studies^31,32^—found that a low number of motor errors did not impair the serial-order-based learning and the acquired knowledge of probability-based regularities as could be predicted by these theories. However, the increased number of motor errors (as in the speed condition in our study) might be advantageous for the initial performance on probability-based regularities, although the advantage disappears when the learning information needs to be accessed under different conditions. Thus, it is possible that the motor errors may not be as important for updating the priors under all circumstances. However, there are arguments against this interpretation. First, the predictive model may be already capable of performing reliable predictions even at an early stage of learning. Second, fewer errors are made by participants who perform worse in RTs, potentially indicating that prediction errors may take on a different form when specific instructions are provided. Third, it is unclear if and how predictions or prediction errors in the brain translate to accuracy and RTs in behavior, as well as whether errors made during a behavioral task are caused by brain predictions or if there are other factors as well. To address these issues, further studies are needed on the relationship between the effect of speed and accuracy instructions on learning mechanisms and prediction errors.

Another potential interpretation of the lower performance of probability-based learning under accuracy instruction could be that participants in this group focused more on correct answering rather than speed, which is reflected in the RT difference scores as the relatively slow responses would result in poorer discrimination between trial types. However, this possibility is not likely, as, in our previous study with the non-cued version, no differences were observed between the groups in learning, although large differences in average RTs were detected^12^. More importantly, the lack of difference between groups after the change of the instructions speaks against the possibility that the lack of differentiation was worse for participants who performed under accuracy instructions. Even if this were true, it does not change our conclusion that only knowledge expression is affected by instructions, but learning was similar for both groups.

Looking at our results from the perspective of the implicitness (incidentality) vs. explicitness (intentionality) of learning, the cueing in the learning task made the structure of the learned sequence (the pattern trial vs. random trial distinction) explicit to the participants. The explicitness of this aspect is clear, as participants were able to report the sequence with high precision on the post-block sequence report task. However, we cannot state that serial-order learning is entirely explicit: although RTs of pattern high-probability trials reflect mostly explicit knowledge, the features of random high-probability trials are acquired through implicit learning. Therefore, higher serial-order-based learning (i.e., the higher difference between the most explicitly and mostly implicitly learned aspects) might indicate more explicit learning than probability-based learning. This assumption is supported by some evidence showing a positive correlation between sequence knowledge and serial-order-based learning^19^. However, other studies show that even if participants can successfully report the sequence on the post-block sequence report task, they do not necessarily show enhanced serial-order-based learning^18,33^. Thus, they cannot use their explicit knowledge to boost performance on the ASRT task. We cannot exclude the possibility that the performance on random high-probability trials is influenced by implicit, incidental processes^18^. Although we did not directly test that, we can assume that probability-based learning is relatively implicit, as participants are not aware of the probabilities of different trials. In this perspective, our results imply that speed-focused instructions selectively affect purely implicit knowledge expression, and more explicit processes are less influenced. Future studies will be required to investigate further how explicitness might influence the effect of speed and accuracy instructions on performance, as explicit and implicit memory processes can occur parallel during skill learning^33^.

Purely explicit knowledge of the sequence was evaluated by the post-block sequence report performance. Here, we found a trend-level advantage for the participants who learned with accuracy instructions. It could mean that instructions to focus on accuracy increase the explicitness of the to-be-learned pattern at the beginning of the task. The reason for this result could be that it is harder to follow cueing at a faster speed, which is consistent with the findings that increasing response-to-stimulus interval is associated with higher explicitness in sequence learning^34^. These results could have an important message from a methodological perspective: future studies may consider giving speed instructions if they aim to keep participants implicit in a particular pattern of learning. We must stress, however, that the speed and accuracy differences were at trend levels; therefore, more studies are needed to draw firm conclusions about the effect of instructions in this regard.

Lastly, as a limitation of our study, we should mention the lack of a control group, which would have helped overcome the potential bias caused by the fact that pattern elements were cued, which would have caused participants to speed-up disproportionately to these elements. However, we do not think that this distorted our results, as the difference was only observed in probability-based learning, where only random (non-cued) elements were compared. Another limitation of the study is that the gender ratios were not equalized in our study: the sample was predominantly female due to the non-uniform availability of female and male participants. To our knowledge, no gender differences in initial ASRT task performance have been reported so far. Because of this, we could not exclude entirely the possibility that the effect of instructions on the two investigated aspects of procedural learning differs according to gender, which would be worthwhile to explore in future research.

Our study investigated the effects of speed and accuracy instructions on two essential aspects of procedural learning, namely, the acquisition of probability-based and serial-order-based regularities. Based on our results, speed and accuracy instructions affect these two aspects of procedural learning differently; although picking up probability-based regularities can be faster with speed instructions, it does not result in more stable acquired knowledge. On the other hand, the learning of serial-order-based regularities seems to be resistant to instructions, thus, to the manipulation of speed/accuracy trade-off. As the performance and retrieval of knowledge are also affected differently in probability-based learning, it draws attention to the differences in competence and performance in the measurement of learning. Moreover, the fact that learning has occurred with almost errorless performance suggests that procedural learning does not depend exclusively on response errors.

## Methods

### Participants

Fifty-six healthy young adults participated in this study. Eight participants were excluded from the analysis because, based on their performance, they did not follow the instructions properly. Six of them were excluded based on their sequence report performance (accuracy below 30%, see details in the *“*Analysis of the post-block sequence report task*”* section), and two of them based on general speed and accuracy (exclusion criteria same as in Vékony et al.^12^). As a result, 48 participants (43 females) remained in the final sample, which is sufficient to detect group differences on the ASRT task. Participants were randomly assigned to one of the two experimental groups: Accuracy Group (*N* = 26) and Speed Group (*N* = 22).

The age of the participants ranged from 18 to 34 years (M_age_ = 21.21 years ± 2.81 SD). All of them were undergraduate students who received course credit for participating (M_education_ = 14.10 years ± 2.01 SD). Their working memory performance was in the normal range, measured by the counting span task (M_Counting Span_ = 3.67 ± 0.98 SD)^35^. Handedness was determined by the laterality quotient based on the Edinburgh Handedness Inventory (N_right-handed_ = 35, N_left-handed_ = 3, N_ambidextrous_ = 10)^36^. No significant group differences were observed in these measures (see Table 1). None of the participants reported a history of neurological or psychiatric disorders. All participants had a normal or corrected-to-normal vision. Written informed consent was obtained from all participants. The study was approved by the Research Ethics Committee of the Eötvös Loránd University, Budapest, Hungary, and was conducted in accordance with the Declaration of Helsinki.

**Table 1:** Characteristics of the two experimental groups.

	Accuracy group (*N* = 26)	Speed group (*N* = 22)	Group comparison
Age (years)	21.54 ± 3.09	20.82 ± 2.46	*p* = 0.38
Education (years)	14.54 ± 2.16	13.60 ± 1.74	*p* = 0.10
Gender (m/f)	2/24	3/19	*p* = 0.50
Handedness (r/l/a)	20/1/5	15/2/5	*p* = 0.70
Counting span	3.53 ± 0.93	3.83 ± 1.03	*p* = 0.29

Means and standard deviations for age, education, and counting span are presented. For gender (*m* male, *f* female) and handedness (*l* left-handed, *r* right-handed, *a* ambidextrous) case numbers per group are presented. Error-values indicate standard deviation.

### Justification of sample size

A power analysis was conducted using G*Power version 3.1.9.7^37^ to determine the minimum sample size required to test our hypotheses. Results indicated the required sample size to achieve 80% power for detecting at the lowest expected effect size of η_p_^2^ = 0.12 as in SPSS^38^ (derived from previous ASRT studies that detected group differences using mixed-design analysis of variance, ANOVA^19,39–41^), at a significance criterion of α = 0.05, was *N* = 30 and *N* = 38 for the between-within interaction and a between-subjects main effect of a 2 × 4 ANOVA, respectively. Thus, the obtained sample size of *N* = 48 is adequate to test the study hypotheses.

### Cued version of the Alternating Serial Reaction Time (ASRT) task

**Fig. 4:**
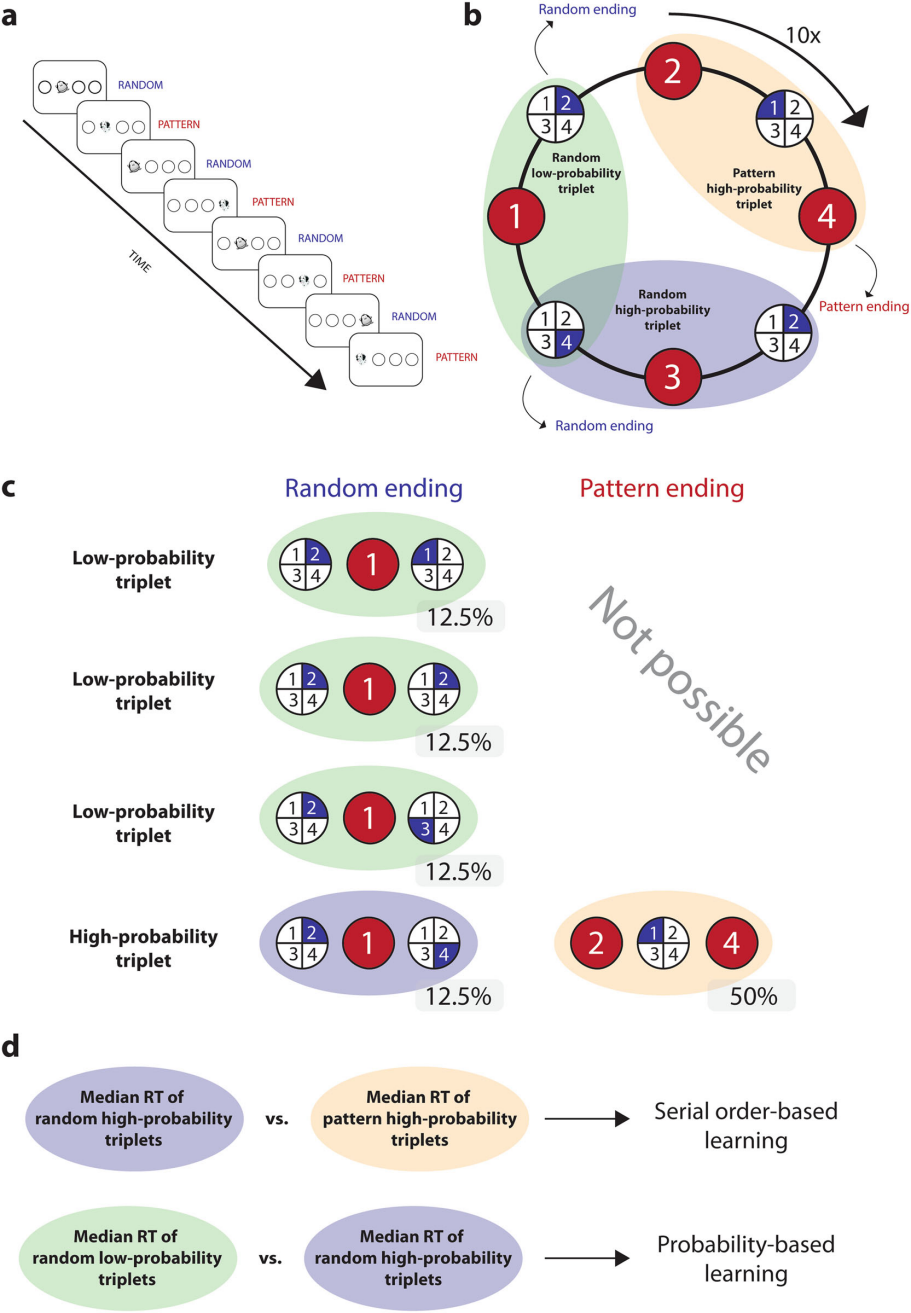
The Alternating Serial Reaction Time (ASRT) task. **a** Visualization of the Alternating Serial Reaction Time task. A drawing of a dog head or a penguin appeared as a target stimulus in one of the four locations. Every other trial (dog) followed a predetermined order of appearance, while the remaining trials (penguin) appeared at randomly selected positions (r). **b** The formulation of triplets in the task. Each trial was categorized as a pattern high-probability trial, a random high-probability trial, or a random low-probability trial based on the positions of the two preceding trials. **c** An example of the different triplet types. Two main cases can occur: the triplet is ending in a pattern trial (50%), or a random trial (50%). If the triplet is ending in a pattern trial, then the n-2 trial is necessarily a pattern trial due to the alternating sequence; therefore, the last element can be at only one particular position, which is defined by the four-element sequence of predetermined elements. If the triplet is ending with a random trial, four cases are possible: after the first two elements, the third element can be in any position. However, one out of these four cases will match the formation that occurs when a triplet ends in a pattern trial. Therefore, it will also be a high-probability triplet. **d** The calculation of the serial-order-based and probability-based learning scores.

We used the cued version of the ASRT task^19^. Four empty black circles appeared horizontally in the middle of the screen (Fig. 4a). The Z, C, B, and M keys on a QWERTY keyboard corresponded to the four circles on the screen. A target stimulus (a drawing of a dog’s head or penguin) appeared in one of the four circles. Participants were instructed to use their middle and index fingers on both hands to indicate the location of the stimuli by pressing the corresponding key on a keyboard. After a correct keypress, the target stimulus disappeared from the screen, and after a 120 ms-long response-to-stimulus interval, a new target appeared in one of the four positions.

The order of appearance of the target stimuli followed an alternating sequence: every other element appeared in a predetermined (pattern) position, and the remaining elements’ positions were randomly chosen from the four possible locations. The predetermined elements followed a four-element sequence. We mark sequence positions from left to the right as 1 to 4; a possible sequence is, for example, 2431. In this case, the alternating sequence was 2r4r3r1r (where *r* indicates a random position). The predefined and random elements result in an alternating eight-element sequence. Each block of ASRT contained ten repetitions of the 8-element sequence preceded by five random trials as warm-up trials. Thus, each block contained 85 trials in total. The participants were permitted to take a short break after each block and continue when they were ready.

The predetermined (pattern) and random elements were visually distinct: pattern elements were marked with dog heads and random ones with penguins. Participants were informed that the dog heads followed a predetermined sequence and that the penguins appeared randomly. They were not informed about the exact sequence but were asked to find the order of appearance of the dogs to improve their performance. For each participant, one of the six unique sequence permutations was selected pseudorandomly. For a given participant, the sequence permutation was the same throughout the experiment.

Because of the alternating sequence of random and pattern trials, some runs of three consecutive trials (referred to as *triplets*) were more probable than others (Fig. 4b). For instance, if the sequence was 2r4r3r1r, triplets such as 2-X-4, 4-X-3, 3-X-1, and 1-X-2 (where X indicates the middle element of a triplet) occured with a higher probability than triplets such as 2-X-1 or 2-X-3 as their first and third elements could be either a pattern or a random trial. We refer to more probable triplet types as high-probability triplets, and less probable ones as low-probability triplets^42^. Please note that the expression triplet refers to three consecutive elements of the sequence (which can be evaluated as a high- or low-probability triplet), whereas the expression trial refers to a single element of the sequence (which can be a pattern or random element, and also, the last element of a high- or low-probability triplet). Sixty-four different triplets could occur during the task. Sixteen of them were high-probability, and 48 of them were low-probability triplets. High-probability triplets can be formed by two pattern trials and one random trial in the middle (50% of all trials) or two random trials and one pattern trial in the middle (12.5% of all trials). Thus, 62.5% of all trials were the last element of a high-probability triplet, and 37.5% of all trials were the last element of a low-probability triplet (Fig. 4c). In summary, three trial types could be differentiated: (1) predetermined (pattern) elements appearing as the last element of a high-probability triplet (henceforth referred to as pattern high-probability trials); (2) random elements appearing as the last element of a high-probability triplet (henceforth referred to as random high-probability trials); and (3) random elements appearing as the last element of a low-probability triplet (henceforth referred to as random low-probability trials).

Random high-probability trials and random low-probability trials share the same sequence properties (neither of them is a predetermined element) but differ in statistical properties as they correspond to the last element of a high-probability or a low-probability triplet, respectively. The learning of probability-based regularities was measured by the difference in RTs between random high-probability and random low-probability trials: faster RTs are expected for random high- vs. low-probability trials. On the other hand, random high-probability and pattern high-probability trials share the same statistical properties (they are both high-probability trials) but differ in sequence properties as they correspond to random or pattern elements, respectively. The learning of serial-order-based regularities was quantified by the difference in RTs between random high-probability vs. pattern high-probability trials: with greater serial-order learning, faster RTs are expected for pattern high-probability vs. random trials (Fig. 4d).

### Post-block sequence report task (within the ASRT task)

The explicit knowledge of participants was tested after every ASRT block. Participants were instructed to type in the order of pattern elements (dog head stimuli) using the four response keys. Each sequence report task lasted until 12 keypresses and was repeated after each block of ASRT^16–18,43^. The reason behind providing 12 button presses was that participants were not informed about the length of the sequence they had to look for. Therefore, we wanted to provide an opportunity for the participants to type longer sequences in their answers. Also, a higher number of keypresses enabled us to differentiate between guesses and valid knowledge.

The task was assessed based on response accuracy (i.e., % of correct keypresses, see details in the “Analysis of the post-block sequence report task” section). For example, if the sequence was 1r2r3r4r, the correct answer would be 1234 four times, that is, 123412341234 (100% correct answer). As the starting position of the sequence does not affect the learned probabilities, the answers 234123412341, 341234123412, and 412341234123 were equally evaluated as 100% correct. The test of explicit knowledge (hereafter referred to as sequence report) was performed in both the Different and Same Instruction phases after each block.

### Study design

**Fig. 5:**
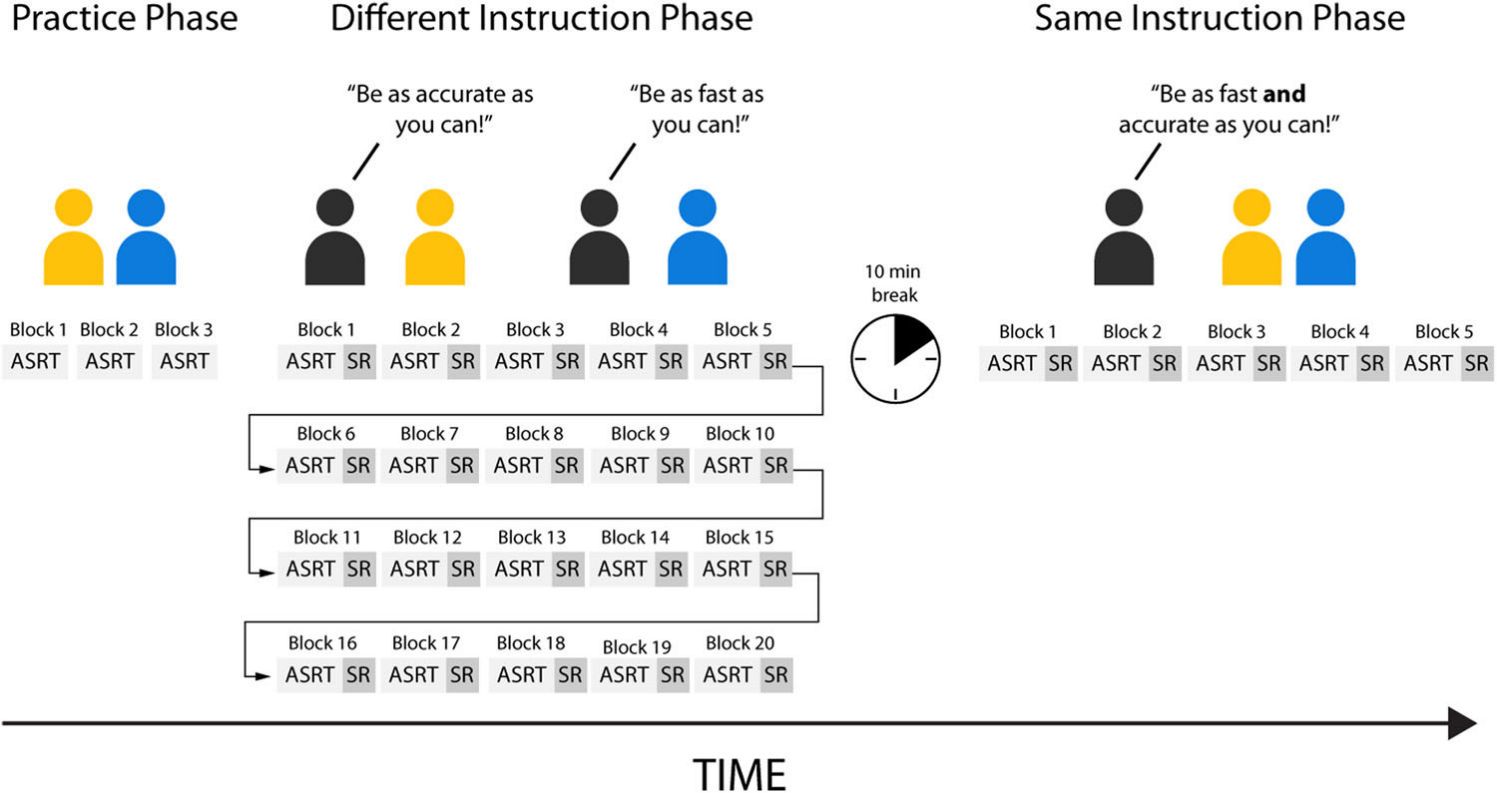
Study design. Participants completed three blocks of practice to familiarize themselves with the task. After that, participants received instructions to either be accurate (Accuracy Group) or fast (Speed Group) during the next phase of the experiment (Different Instruction Phase). Moreover, they were told that specific stimuli (the dogs) followed a predetermined sequence, and they should use this information to improve their performance. Participants completed 20 blocks of the ASRT task. After each block ASRT task, participants had to perform the sequence report task (SR). After the Different Instruction Phase, participants rested for 10 min. Next, during the Same Instruction Phase, the participants’ tasks remained the same, but this time, they were instructed to be equally fast and accurate.

First, participants completed three practice blocks of the ASRT task to familiarize themselves with the response keys; all presented stimuli were random during practice. The participants then completed 20 blocks of the ASRT task (1 block = ~1–1.5 min). The Accuracy Group was instructed to perform the task as accurately as possible; the Speed Group was told to be as fast as possible during the task (Different Instruction Phase). After 20 blocks, participants rested for 10 min. After that, the participants performed another set of five blocks of the ASRT task. However, this time, they were instructed to focus equally on speed and accuracy (Same Instruction Phase) (Fig. 5). In both phases, after each block, the post-block sequence report task was performed.

### Analysis of the ASRT task

Preprocessing of the ASRT task was performed following standard methods^42,44^. We collapsed the blocks of the ASRT task into five analysis units (epochs). Thus, the first epoch contained blocks 1–5, and the second epoch contained blocks 6–10, and so forth. Therefore, the Different Instruction Phase contained four epochs, and the Same Instruction Phase contained one epoch.

Each trial was categorized as the third element of a random high-probability, pattern high-probability, or random low-probability triplet (i.e., the probability of each trial was evaluated based on the position of the previous two trials). Trials with inaccurate responses, trials that were the last elements of trills (e.g., 1-2-1), and repetitions (e.g., 1-1-1) were excluded from the analysis, as participants typically show pre-existing tendencies toward them^20^ (see analysis without excluding trills and repetitions in “Analysis without excluding trills and repetitions” section of the Supplementary Materials). Median RT were calculated separately for the three trial types in each epoch. As a substantial ceiling effect occurs with accuracy instructions during the ASRT task^12^, we only considered RTs for analysis.

Based on these three types of trials, scores for serial-order- and probability-based learning can be quantified^19^. The learning of probability-based regularities was defined as the difference in median RTs between random high- and low-probability trials. The learning of serial-order-based regularities was defined as the difference in median RTs between pattern high-probability and random high-probability trials. As a result of the speed/accuracy instructions, RTs were highly different between the two groups (see section “Did the instructions change overall reaction time and accuracy?”). To ensure that the potential differences in learning were not due to significant differences in RT, we divided the learning scores by the median RT of the given epoch. Non-standardized results can be found in the Supplementary Materials (“Results of analyses with non-standardized learning scores” section) as well as visualization of the three trial types (random high-probability, random low-probability, and pattern high-probability trials) and the average RTs (“Visualization of trial types and average RTs” section).

Statistical analysis was performed using JASP 0.16^45^. We used mixed-design ANOVAs to compare general speed and accuracy changes, probability-based and serial-order-based learning of the two groups in the Different Instruction Phase (see “Comparison of probability-based vs. serial-order-based learning” section of the Supplementary Materials for a comparison of the two learning types). For the ANOVAs, corrected *df* values and corrected *p* values are reported (if applicable) along with partial eta-squared (η_p_^2^) as the measure of effect size. Mann–Whitney U tests were used to compare the performance of the practice phase, as well as the learning of serial-order-based and probability-based regularities in the Same Instruction Phase. Rank-Biserial Correlation (r_RB_) values are reported as effect size measures for the Mann–Whitney U tests.

In addition to the frequentist approach, Bayesian ANOVAs and Mann–Whitney U tests were performed with similar parameters. Here, we present the results of Bayesian Model Averaging and report the BF exclusion (*BF*_exclusion_) values (1/*BF*_inclusion_) along with frequentist statistics. *BF*_exclusion_ values indicate the amount of evidence for excluding a given factor from our model. Thus, values below 1 support inclusion, and values above 1 support exclusion of the given factor. Full model comparisons are included in the Supplementary Materials (see “Bayesian Model Comparisons” section in the Supplementary Materials). Cauchy prior distribution was used for the ANOVA with a fixed-effects scale factor of *r* = 0.5, and a random-effects scale factor of *r* = 1 (JASP Team, 2021). Bayesian Mann–Whitney U tests were performed using the default prior distribution (*r* = 0.707).

### Analysis of the post-block sequence report task

After each block of the ASRT task, the sequence report task was performed by the participants. The participants were instructed to type in the order of pattern elements (dog head stimuli), using response keys, up to 12 keypresses. The accuracy of sequence reports was calculated separately for each block. Each keypress was scored as correct or incorrect relative to the position of the previous keypress. We then calculated the percentage of correct keypresses. Here, the possible values ranged from 0 to 100% (0% = no key press was correct after the previous key press; 100% = all key presses were correct after the previous key press). Thus, an accuracy of 100% indicated that the series of keypresses perfectly reflected the sequence embedded in the ASRT task. Then, the 20 block-wise percentage scores of the Different Instruction Phase and the five percentage scores of the Same Instruction Phase were calculated and averaged according to the four epochs (blocks 1–5 for Epoch 1, blocks 6–10 for Epoch 2, etc.). A higher explicit knowledge percentage score reflects a more stable explicit knowledge of the sequence structure.

To evaluate how explicit sequence knowledge emerged during the task, a mixed-design ANOVA was run on epoch-wise explicit sequence report scores. The same between and within-subject factors were used for the ANOVA run on the ASRT task. To compare explicit sequence knowledge in the Same Instruction Phase, Mann–Whitney U tests were run on the sequence report performance with parameters similar to those of the ASRT learning scores.

## Supplementary information

Supplementary Material

## Data Availability

All data files are openly available from the OSF database: https://osf.io/h6s2t/.
