# Supplementary material for: Speed and accuracy instructions affect two aspects of skill learning differently
Source: NPJ Sci Learn. 2022 Oct 22;7:27. doi: 10.1038/s41539-022-00144-9 (PMC9588023; doi:10.1038/s41539-022-00144-9)
Supplement: Supplementary file 1 — Supplementary Material [file 41539_2022_144_MOESM1_ESM.pdf]

Supplementary Materials

Speed and accuracy instructions affect two aspects of skill learning differently

Teodóra Vékony[1], Claire Pleche[1,2], Orsolya Pesthy[3,4], Karolina Janacsek[4,5], Dezso Nemeth[1,4,6*]

[1]Lyon Neuroscience Research Center (CRNL), INSERM U1028, CNRS UMR5292, Université Claude Bernard Lyon 1, Lyon, France

[2]Département d'Études Cognitives, École Normale Supérieure, Université PSL, 75005, Paris, France

[3]Doctoral School of Psychology, ELTE Eötvös Loránd University, Budapest, Hungary

[4]Institute of Psychology, ELTE Eötvös Loránd University, Budapest, Hungary

[5]Centre of Thinking and Learning, Institute for Lifecourse Development, School of Human Sciences, Faculty of Education, Health and Human Sciences, University of Greenwich, London, United Kingdom

[6]Brain, Memory and Language Research Group, Institute of Cognitive Neuroscience and Psychology, Research Centre for Natural Sciences, Budapest, Hungary

* Corresponding author. Lyon Neuroscience Research Center (CRNL), INSERM U1028, CNRS UMR5292, Université Claude Bernard Lyon 1, Centre Hospitalier Le Vinatier, Bâtiment 462, Neurocampus 95 boulevard Pinel 69675 Bron, France. E-mail address: dezso.nemeth@univ-lyon1.fr (D. Nemeth).

Analysis without excluding trills and repetitions

Here, all the analysis was performed as described in the main article, except that trills and repetitions were not excluded.

Did the instructions affect the learning of probability-based regularities in the Different Instruction Phase?

Standardized probability-based learning scores were compared between the groups in the Different Instruction Phase. Overall, Speed Group showed larger learning scores compared to the Accuracy Group [$M_{\text{Accuracy Group}}$ = 0.03 ± 0.01 SE, $M_{\text{Speed Group}}$ = 0.06 ± 0.01 SE, main effect of Group: $F(1, 46) = 5.63$, $p = .02$, $\eta_p^2 = .11$, $BF_{\text{exclusion}} = 0.68$]. No main effect of Epoch was found, $F(1.54, 70.96) = 0.84$, $p = .41$, $\eta_p^2 = .05$, $BF_{\text{exclusion}} = 12.92$, and the interaction between Epoch and Group was non-significant, $F(1.54, 70.96) = 1.20$, $p = .30$, $\eta_p^2 = .03$, $BF_{\text{exclusion}} = 4.51$.

Did the instructions affect knowledge of probability-based regularities in the Same Instruction Phase?

In the Same Instruction Phase, standardized probability-based learning scores were compared between the groups in the Same Instruction Phase. Contrary to the results of the Different Instruction Phase, no difference was found between groups in the Same Instruction Phase, $U = 263$, $p = .64$, $r_{\text{RB}} = -.08$, $BF_{01} = 3.26$.

Did the instructions affect the learning of serial order-based regularities in the Different Instruction Phase?

Standardized serial order-based learning scores were compared between the groups in the Different Instruction Phase. The two groups showed similar level of serial order-based learning in the Different Instruction Phase [$M_{\text{Accuracy Group}}$ = 0.03 ± 0.01 SE, $M_{\text{Speed Group}}$ = 0.02 ± 0.01 SE, main effect of Group: $F(1, 46) = 0.09$, $p = .76$, $\eta_p^2 = .002$, $BF_{\text{exclusion}} = 5.02$]. No main effect of Epoch was found, $F(1.50, 68.84) = 0.50$, $p = .56$, $\eta_p^2 = .01$, $BF_{\text{exclusion}} = 22.33$, and the interaction between Epoch and Group was non-significant, $F(1.50, 68.84) = 1.26$, $p = .28$, $\eta_p^2 = .03$, $BF_{\text{exclusion}} = 4.12$, indicating that the trajectory of learning was also similar.

Did the instructions affect knowledge of serial order-based regularities in the Same Instruction Phase?

Standardized serial order-based learning scores were compared between the groups in the Same Instruction Phase. Similar to the results of the Different Instruction Phase, no significant difference was found between the two groups in the Same Instruction Phase, $U = 295$, $p = .86$, $r_{\mathrm{RB}} = .03$, $BF_{01} = 3.28$.

Results of analyses with non-standardized learning scores

Here, all the analysis was performed as described in the main article, except that learning scores were not standardized.

Did the instructions affect the learning of probability-based regularities in the Different Instruction Phase?

Non-standardized probability-based learning scores were compared between the groups in the Different Instruction Phase. No overall difference was detected between groups [$M_{\text{Accuracy Group}}$ = 15.45 , $M_{\text{Speed Group}}$ = 21.50, main effect of Group: $F(1, 46) = 2.455$, $p = .12$, $\eta_p^2 = .05$, $BF_{\text{exclusion}} = 1.46$]. A main effect of Epoch was found, $F(3, 138) = 3.11$, $p = .03$, $\eta_p^2 = .06$, $BF_{\text{exclusion}} = 0.69$. However, the Bonferroni-corrected post-hoc comparisons did not reveal significant differences between epochs, only a trend-level difference was found between the first and second epoch ($p = .06$, all other ps > .20). The interaction between Epoch and Group was found to be significant, $F(3, 138) = 2.98$, $p = .03$, $\eta_p^2 = .06$, $BF_{\text{exclusion}} = 0.70$ (Supplementary Figure 1). The post-hoc comparisons revealed a significant difference between groups in the third epoch ($p = .03$).

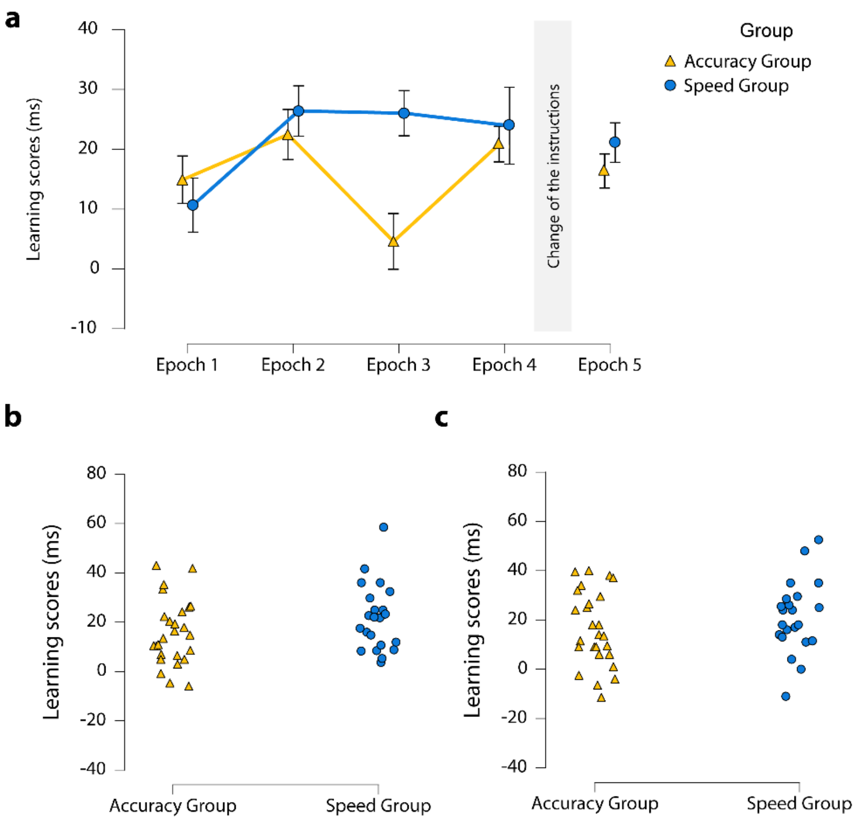

Supplementary Figure 1. Learning of probability-based regularities. (a) The dynamics of learning of probability-based regularities with accuracy or speed instructions. The y-axis represents the standardized learning scores [(random low-probability trials – random high-probability trials in the given epoch)/median RT of the given epoch], and the x-axis of the five epochs (the first four are of the Different Instruction Phase, and the fifth one is of the Same Instruction Phase). The Accuracy Group is presented with yellow, while the Speed Group with blue color. Error bars represent the standard error of the mean. In the Different Instruction Phase, the Speed Group shows an advantage of learning, but it disappears in the Same Instruction Phase. **(b) Individual data of the significant main effect of Group of probability-based learning in the Different Instruction Phase.** Triangles and dots represent the individual data points. **(c) Individual data of the lack of significant main effect of Group of probability-based learning in the Same Instruction Phase.** Triangles and dots represent the individual data points.

Did the instructions affect knowledge of probability-based regularities in the Same Instruction Phase?

In the Same Instruction Phase, the non-standardized probability-based learning scores were compared between groups. Contrary to the results of the Different Instruction Phase, no difference was found between groups in the Same Instruction Phase, $U = 235$, $p = .30$, $r_{RB} = -.18$, $BF_{01} = 2.30$ (Supplementary Figure 1).

Did the instructions affect knowledge of serial order-based regularities in the Different Instruction Phase?

Non-standardized serial order-based learning scores were compared between the groups in the Different Instruction Phase. The two groups showed equal serial order-based learning in the Different Instruction Phase [$M_{\text{Accuracy Group}} = 11.32$ ms, $M_{\text{Speed Group}} = 6.08$ ms, main effect of Group: $F(1, 46) = 1.24$, $p = .27$, $\eta_p^2 = .03$, $BF_{\text{exclusion}} = 3.76$]. No main effect of Epoch was found, $F(2.53, 116.51) = 0.12$, $p = .93$, $\eta_p^2 = 0.003$, $BF_{\text{exclusion}} = 45.40$, and the interaction between Epoch and Group was non-significant, $F(2.53, 116.51) = 1.51$, $p = .22$, $\eta_p^2 = .03$, $BF_{\text{exclusion}} = 96.82$ (Supplementary Figure 2).

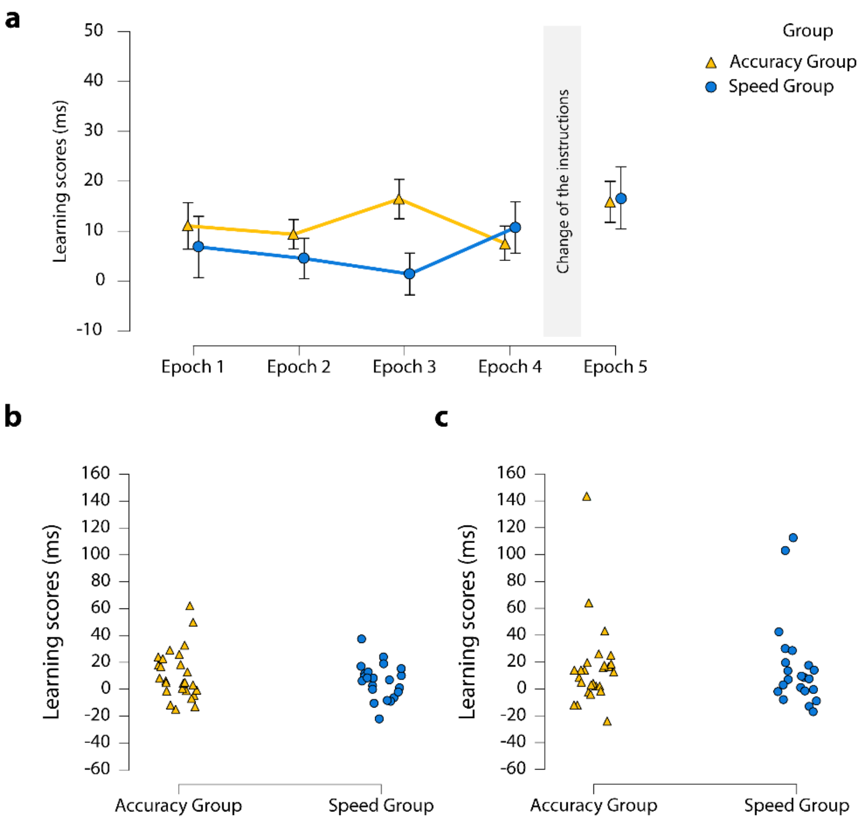

Supplementary Figure 2. Learning of serial order-based regularities. (a) The dynamics of learning of serial order-based regularities with accuracy or speed instructions. The y-axis represents the standardized learning scores [(random low-probability trials – random high-probability trials in the given epoch)/median RT of the given epoch], and the x-axis of the five epochs (the first four are of the Different Instruction Phase, and the fifth one is of the Same Instruction Phase). The Accuracy Group is presented with yellow, while the Speed Group with blue color. Error bars represent standard error of the mean. Both groups show equal learning in both phases. **(b) Individual data of the significant main effect of Group of serial order-based learning in the Different Instruction Phase.** Triangles and dots represent the individual data points. **(c) Individual data of the lack of significant main effect of Group of serial order-based learning in the Same Instruction Phase.** Triangles and dots represent the individual data points.

Did the instructions affect knowledge of serial order-based regularities in the Same Instruction Phase?

Non-standardized serial order-based learning scores were compared between groups in the Same Instruction Phase. Similar to the results of the Different Instruction Phase, no

difference was found in learning between groups in the Same Instruction Phase, $U = 302$, $p = .75$, $r_{RB} = .06$, $BF_{01} = 3.29$ (Supplementary Figure 2).

Visualization of trial types and average RT

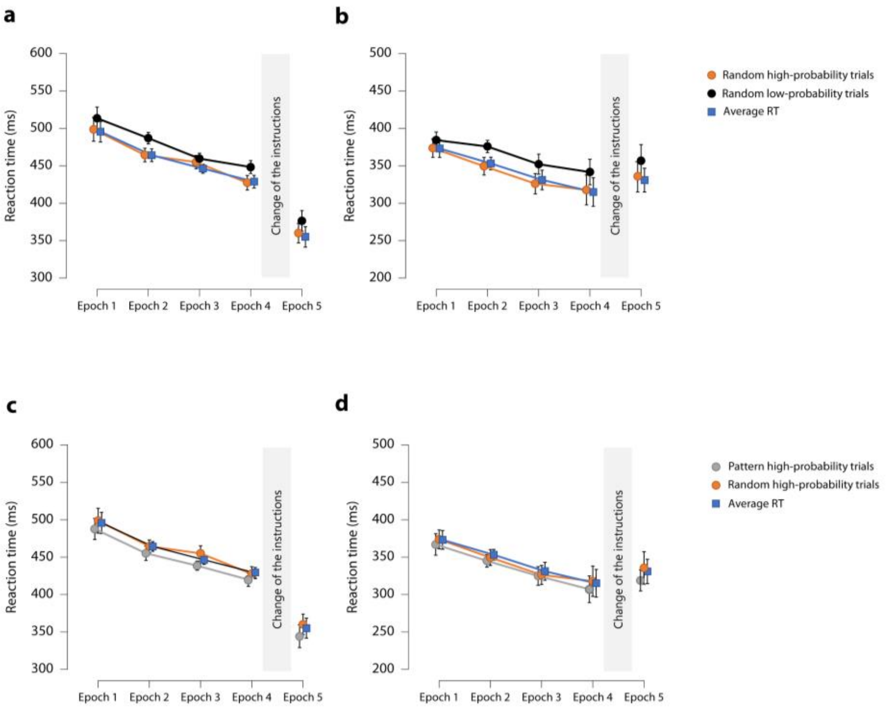

Supplementary Figure 3. Visualization of the three trial types and the average RT. (a) The trials used for the calculation of probability-based learning and average RT in the Accuracy Group and (b) the Speed Group. c) The trials used for the calculation of serial order-based learning and average RT in the Accuracy Group and (d) the Speed Group. Please note that the scaling of the y-axis is different for the two groups for visualization purposes. The error bars indicate 95% confidence intervals.

Comparison of probability-based vs. serial-order based learning

How does probability-based learning and serial order-based learning compare in the Different Instruction Phase?

We have performed a Epoch (1-4) × Learning Type (probability-based vs. serial order-based) × Group (Accuracy Group vs. Speed Group) mixed-design ANOVA for the Different Instruction Phase:

We found a main effect of Epoch, $F(1.77, 72.83) = 3.46$, $p = .04$, $\eta_p^2 = .07$, $BF_{exclusion} = 9.96$, indicating that learning changed between epochs. Bonferroni-corrected post-hoc tests revealed that a significant improvement were detected between the first and last epoch ($p = .01$); however, the Bayesian analysis spoke against this difference. The Epoch by Group interaction was not significant, $F(1.77, 72.83) = 1.87$, $p = .16$, $\eta_p^2 = .04$, $BF_{exclusion} = 11.58$, indicating that the change of learning thoughout the task did not differ between groups. We found a main effect of Learning Type, $F(1, 46) = 11.63$, $p = .001$, $\eta_p^2 = .20$, $BF_{exclusion} = 0.04$, revealing that probability-based learning was overall higher than serial order-based learning ($M_{probability\text{-}based\ learning} = 0.05$, SE = 0.01; $M_{serial\ order\text{-}based\ learning} = 0.02$, SE = 0.01). The Learning Type by Group interaction was also significant, $F(1, 46) = 5.66$, $p = .02$, $\eta_p^2 = .11$, $BF_{exclusion} = 0.36$. Bonferroni-corrected post-hoc test revealed that probability-based learning was higher in the Speed Group than in the Accuracy Group ($p = .03$), however, serial order-based learning did not differed between the two groups ($p > .99$). The Learning Type by Epoch interaction was not significant, $F(1, 46) = 5.66$, $p = .55$, $\eta_p^2 = .11$, $BF_{exclusion} = 14.33$, indicating that the change between epochs was similar between th two types of learning. The Learning Type by Epoch by Group interaction also remained non-significant, $F(1.58, 72.83) = 1.22$, $p = .29$, $\eta_p^2 = .03$, $BF_{exclusion} = 2.27$, indicating that the (lack) of interaction was not different by groups. The Group main

effect approached significant, $F(1, 46) = 3.04$, $p = .09$, $\eta_p^2 = .06$, $BF_{\text{exclusion}} = 2.27$, but Bayesian analysis did not seem to support the overall group difference.

How does probability-based and serial order-based knowledge compare in the Same Instruction Phase?

We have performed a Learning Type (probability-based vs. serial order-based) × Group (Accuracy Group vs. Speed Group) mixed-design ANOVA for the Same Instruction Phase:

No difference was found in Learning Type, $F(1, 46) = 0.09$, $p = .77$, $\eta_p^2 = .002$, $BF_{\text{exclusion}} = 4.50$, indicating that similar level of acquired knowledge was measured in the two types of learning in the Same Instruction Phase. The Learning by Group interaction was also non-significant, $F(1, 46) = 0.03$, $p = .86$, $\eta_p^2 < .001$, $BF_{\text{exclusion}} = 3.33$, indicating a lack of difference of the acquired probability-based vs. serial-order based knowledge between groups. The Group main effect was also non-significant, $F(1, 46) = 0.68$, $p = .41$, $\eta_p^2 = .02$, $BF_{\text{exclusion}} = 2.98$, revealing a lack of overall difference between the groups in acquired knowledge.

Bayesian Model Comparisons

Probability-based learning (Different Instruction Phase)

Supplementary Table 1. Bayesian model comparisons for probability-based learning of the Different Instruction Phase

Models	P(M)	P(M|data)	BF_M	BF_{01}	error %
Null model (incl. subject)	0.200	0.177	0.862	1.000	
Group	0.200	0.497	3.952	0.357	0.770
Epoch + Group	0.200	0.193	0.959	0.917	1.353
Epoch + Group + Epoch ✻ Group	0.200	0.067	0.289	2.634	1.351
Epoch	0.200	0.065	0.278	2.733	0.531

Note. All models include subject. The Models column indicates the predictors included in each model, the P(M) column the prior model probability, the P(M|data) column the posterior model probability, the BF_M column the posterior model odds, and the BF_{01} column the Bayes factors of all models compared to the null model. The error is an estimate of the numerical error in the computation of the Bayes factor.

Serial order-based learning (Different Instruction Phase)

Supplementary Table 2. Bayesian model comparisons for serial order-based learning of the Different Instruction Phase

**Models**	**P(M)**	**P(M|data)**	**BF_M**	**BF_{01}**	**error %**
Null model (incl. subject)	0.200	0.797	15.671	1.000	
Group	0.200	0.160	0.761	4.984	0.907
Epoch	0.200	0.034	0.143	23.093	0.791
Epoch + Group	0.200	0.007	0.029	110.487	2.169
Epoch + Group + Epoch ✻ Group	0.200	0.002	0.007	442.628	4.850

Note. All models include subject. The Models column indicates the predictors included in each model, the P(M) column the prior model probability, the P(M|data) column the posterior model probability, the BF_M column the posterior model odds, and the BF_{01} column the Bayes factors of all models compared to the null model. The error is an estimate of the numerical error in the computation of the Bayes factor.
